# Effectiveness of Multicomponent Exercise Interventions in Older Adults With Dementia: A Meta-Analysis

**DOI:** 10.1093/geront/gnaa091

**Published:** 2020-07-11

**Authors:** Flávia Borges-Machado, Nádia Silva, Paulo Farinatti, Roberto Poton, Óscar Ribeiro, Joana Carvalho

**Affiliations:** 1 CIAFEL—Research Centre in Physical Activity, Health and Leisure, Porto, Portugal; 2 Faculty of Sport, University of Porto, Portugal; 3 Laboratory of Physical Activity and Health Promotion, University of Rio de Janeiro State, Brazil; 4 Graduate Program in Physical Activity Sciences, Salgado de Oliveira University, Niteroi, Brazil; 5 Centro Universitário IBMR, Laureate International Universities, Rio de Janeiro, Brazil; 6 CINTESIS—Center for Health Technology and Services Research, Porto, Portugal; 7 Department of Education and Psychology, University of Aveiro, Portugal

**Keywords:** Cognition, Exercise/physical activity, Function/mobility, Intervention, Neurocognitive disorder

## Abstract

**Background and Objectives:**

Multicomponent training (MT) combines aerobic, strength, postural, and balance exercises and may be a promising intervention strategy for dementia. This meta-analysis study aims to systematize evidence concerning the effectiveness of MT in physical fitness, cognition, and functionality on activities of daily living (ADL) in older adults with dementia and to identify moderation patterns regarding training variables.

**Research Design and Methods:**

4 databases were systematically searched to locate potential trials through March 2019. A total of 2,312 records were identified and a final set of 17 manuscripts reviewed; of these, 6 satisfied all eligibility criteria.

**Results:**

Samples sizes ranged from 27 to 170 participants; MT programs lasted between 4 weeks and 12 months, took place from a daily basis to twice a week, and sessions ranged from 30 to 60 min. The TESTEX scale was used to analyze the methodological quality, and the funnel plots to assess the risk of bias. This meta-analysis revealed that MT interventions benefit older adults with dementia regarding ADL performance (effect size = 0.313 [0.16–0.46]; *p* < .01), but the evidence was not sufficiently robust to determine the effectiveness of MT on cognitive function and physical fitness, particularly, on agility.

**Discussion and Implications:**

MT may be an important nonpharmacological strategy to enhance ADL functionality on older adults with dementia. Findings suggest that long-term interventions are more prevalent than high-frequency and longer duration exercise sessions. Further evidence is needed for acknowledging its benefits in specific cognitive abilities and physical fitness. This meta-analysis is registered in PROSPERO (no. CRD42020141545).

Dementia and neurocognitive disorder are umbrella terms used to describe a set of diseases that progressively affect the brain and several cognitive functions (American Psychiatric Association, 2013; World Health Organization [WHO], 2017). In 2012, the WHO placed this condition as a global public health priority (Alzheimer’s Disease International, 2018). Approximately 5% of the world’s older adult population (roughly 47 million people) was affected by dementia in 2015, and estimations predict a total of 75 million in 2030 and 132 million by 2050 (WHO, 2017).

Globally, dementia represents one of the major causes of disability and dependence among older adults (WHO, 2017, 2018, 2019). The *Lancet Commission on Dementia Prevention, Intervention and Care* (2017) has recently emphasized that effective interventions to delay or prevent dementia cases should address multiple reversible risk factors like physical inactivity, depression, metabolic and cardiovascular disease (diabetes, hypertension, and obesity), hearing loss, smoking, social isolation, and poor education (Livingston et al., 2017).

Physical inactivity accounts for 3.8% of cases of dementia worldwide (Sallis et al., 2016) and is the highest (of seven potentially modifiable) population-attributable risk factor (Norton et al., 2014)—between diabetes mellitus, midlife hypertension and obesity, depression, smoking, and low educational attainment. It is estimated that around 3 million cases of Alzheimer’s Disease (AD), the most common dementia type, could be avoided with a 10%–25% shift in modifiable risk factors (Blondell et al., 2014; Erickson et al., 2012). Blondell et al. (2014) emphasized that higher levels of physical activity were associated with an 18% reduction in the risk of dementia.

Physical activity is defined as any corporal movement produced by skeletal muscle contraction resulting in energy expenditure. Exercises refer to planned, structured, and systematic physical activity, with the purpose to maintain or improve at least one of the physical components of physical fitness—aerobics, strength, flexibility, or coordination/balance (American College of Sports Medicine, 2017; Caspersen et al., 1985). Physical activity, and particularly exercise, might have a significative impact to improve cognition and/or prevent dementia (Erickson et al., 2019; Quaglio et al., 2016; Winblad et al., 2016).

The accumulated evidence acknowledges the benefits of exercise as a preventive measure against dementia (Alty et al., 2020; Gomes-Osman et al., 2018; Livingston et al., 2017; WHO, 2019), particularly AD (Norton et al., 2014; Radak et al., 2010). However, prior reviews suggest that the dose–response relationship of interventions to induce those benefits remains undefined (Cass, 2017; Kivipelto et al., 2018; Skinner et al., 2018).

Regular exercise appears to benefit individuals diagnosed with dementia due to its potential influence on treating the symptoms or delaying its progression, in addition to its intrinsic benefits upon physical fitness, cardiovascular health, and individual wellness (Almeida et al., 2019; Cass, 2017; Skinner et al., 2018; Smith et al., 2010). In fact, there is strong evidence that different modalities of exercise (e.g., aerobics, balance, strength training, or a combination of these) may help to delay functional and cognitive decline, minimize the risk of falls, manage neuropsychiatric symptoms, and improve activities of daily living (ADL) independence and quality of life in older individuals with cognitive impairment (American College of Sports Medicine, 2017; Forbes et al., 2015).

Forbes et al. (2015) and Skinner et al. (2018) reinforced the need to identify the best combination of training variables (frequency, intensity, type, and time—or *FITT*) appropriate to a specific type/severity of disease and considering the right target outcome. In fact, the therapeutic role of exercise as a nonpharmacological adjuvant treatment of patients diagnosed with dementia needs further evidence (Livingston et al., 2017).

This meta-analysis focuses on a specific training methodology entitled Multicomponent Training (MT). It has been suggested that MT combining aerobic, strength, postural, and balance exercises (Baker et al., 2007) can improve functional and cognitive performances in healthy older adults (Baker et al., 2007; Carvalho et al., 2009; Toraman et al., 2004) and seems to be a feasible intervention for older adults with dementia (Borges-Machado et al., 2019; Kirk-Sanchez & McGough, 2014; Sampaio et al., 2019, 2020; Smith et al., 2010). These components must be combined during each exercise session and distributed over time along with training planning. Despite multimodal methodologies having been presented as feasible, and to provide a maximum benefit on several dimensions for individuals with dementia (Burton et al., 2015; Hernandez et al., 2015; Lam et al., 2018), the existing systematic reviews and meta-analytical studies regarding the efficacy of exercise for people with dementia do not focus on this specific training methodology (Blankevoort et al., 2010; Farina et al., 2014; Forbes et al., 2015; Gomes-Osman et al., 2018; Groot et al., 2016; Heyn et al., 2004; Lam et al., 2018; Pitkälä et al., 2013; Smith et al., 2010).

In short, stronger evidence is warranted to confirm the role of MT in the improvement of cognitive function, ADL functionality, and physical fitness of older adults with dementia. Moreover, we could not find prior studies investigating the role of FITT components within MT as moderators of changes in cognitive and functional conditions.

To address this gap in the literature, the present meta-analysis includes controlled trials of MT interventions for older adults diagnosed with dementia considering the two following objectives: (a) to determine MT effectiveness to improve physical fitness, cognition, and ADL functionality and (b) to identify moderation patterns of those effects in regard to FITT training variables.

## Method

This meta-analysis is consistent with the Preferred Reporting Items for Systematic Reviews and Meta-Analyses (PRISMA) Statement (Moher et al., 2015) and was registered in the International Prospective Register of Systematic Reviews (registration no. CRD42020141545)—available on request. The PRISMA checklist can be found in Supplementary Table 1.

### Study Inclusion Criteria

The systematic review included studies published in English which meet the following criteria: (a) controlled trials (randomized or not); (b) exercise interventions conducted with older adults clinically diagnosed with dementia (from mild to severe stages); (c) studies performed with humans, with a mean age older than 65 years; (d) interventions applying exclusively MT; (e) peer-reviewed studies. In brief, only trials investigating the effect of MT versus nonexercise control groups on physical fitness, cognitive function, or ADL functionality were included. There was no restriction on intensity, frequency, and duration of exercise programs. Studies exclusively performed with participants diagnosed with Parkinson’s disease, Huntington’s disease, or other rare forms of dementia were excluded, because these patients tend to exhibit significant motor/functional limitations.

### Literature Search Strategy

Potential trials were identified through an electronic literature search using PubMed, Web of Science, SCOPUS (including Embase), and SportDiscus databases. The first two authors (F. Borges-Machado and N. Silva) systematically searched the databases to locate potential trials through March 2019. A Boolean search strategy using the following combination of medical subject heading descriptors and their synonyms was applied: “exercise,” “motor activity,” “circuit-based,” “dementia,” “neurocognitive (disorder/disease),” “cognition,” “executive function,” “activities of daily living,” “functional (independence/performance/autonomy),” “physical (fitness/conditioning),” as presented in Supplementary Table 2.

### Study Selection

The same two authors (F. Borges-Machado and N. Silva) further screened all potential reports using a multistage review process: (a) by title only, (b) by title and abstract, and (c) finally by full-text review. This process was completed using a spreadsheet program from Microsoft. After a detailed review of the articles, F. Borges-Machado conducted manual searches in order to identify additional studies that met the inclusion criteria by consulting the references list of the included studies. Any disagreements regarding potential inclusion or exclusion of studies were resolved by discussion between the authors to achieve consensus. Interrater reliability regarding study selection was assessed by means of Cohen’s kappa, reporting a substantial agreement *k* = 0.742 (*p* < .002); 95% CI [0.41–1.08].

### Data Extraction, Quality Assessment, and Study Outcomes

Data from studies were extracted independently by one author (F. Borges-Machado) according to the following categories: (a) study identification—authors, publication year, country, study design; (b) sample characteristics—size, sex proportion and age, type of dementia, clinical dementia diagnosis criteria, scores in the Mini-Mental State Examination (MMSE), and/or Clinical Dementia Rating; (c) ADL functionality (basic and instrumental ADL measured through validated questionnaires), cognitive function, and physical fitness (gait speed, agility, balance, upper and lower body strength, flexibility, cardiorespiratory capacity, and motor hand function) outcomes with a level of significance; (d) control group treatment; and (e) exercise intervention—FITT variables, intensity monitoring, supervision, and design setting. Corresponding authors were contacted to request additional data/information when required.

As presented in Supplementary Table 3, study quality was assessed by means of the TESTEX scale (Smart et al., 2015), a 12-point checklist with higher scores (maximum 15 points) indicating better study quality.

### Effect Size Estimate

The Hedges’ *g* effect size was used to quantify alterations on cognition, ADL functionality, and physical fitness (agility) from multicomponent interventions, defined as corrected standardized mean difference (SMD) between two groups based on the pooled, weighted standard deviation (SD; Durlak, 2009).

Initially, the paired difference (mean experimental group – mean control group) and paired difference standard deviation (experimental *SD*^2^ + control *SD*^2^ – 2 × intertrial correlation × experimental *SD* × control *SD*)^1/2^ were calculated. Subsequently, we determined the SMD (paired difference × (2 – 2 × intertrial correlation))^1/2^ ÷ (paired difference *SE*) and the SMD standard error ((1/*n* + SMD^2^ ÷ (2 × *n*))^1/2^ × (2 – 2 × correlation factor))^1/2^.

Thus, the correction factor obtained by equation 1 *–* {3 ÷ [4 × (total *n* – 2) –1]} was multiplied by the SMD to estimate the Hegde’s *g* (Borenstein et al., 2009). When studies reported only the *SE* value, the *SD* was calculated by multiplication of *SE* by the square root of the sample *n*. The interstudy correlation factor (correlation between data from experimental and control groups) was not provided by any of the studies, therefore the value an SMD of 0.5 was considered across studies. A positive value of Hedges’ *g* effect size (ES) indicates that the experimental group was superior on a positively oriented outcome measure (ADL functionality, physical fitness, or cognition), when comparing to the control group (Durlak, 2009).

Sensitivity analysis was performed to confirm if the calculated effect sizes of the included studies were dependent on each other (Becker, 1988; Card, 2011).

The *Cochran Q* was calculated to analyze whether the individual studies’ treatment effects are farther away from the common effect, beyond what is expected by chance, that is, to verify if the homogeneity of the observed effect sizes was significant. The *Cochran Q* was converted to a standardized homogeneity measure (*I*^2^ statistic) and to the correspondent confidence interval (95% CI) to evaluate how much heterogeneity was present on the included sample—ranging from 0% to 100%, representing total variation across studies—with values of 25%, 50%, and 75% corresponding to low, moderate, and high heterogeneity (Higgins et al., 2003). As the *I*^2^ approaches 100% and CI do not include 0%, the hypothesis of homogeneity is rejected and it is more probable that heterogeneity has occurred.

### Statistical Analysis

The meta-analysis and meta-regressions were executed through the *Comprehensive Meta-Analysis* program (version 2.2, Biostat Inc., Englewood, NJ). The random-effect model was applied based on the possibility of the samples presenting unknown particularities that could interfere with the true effect of the intervention. Differences between variables of the subgroup were tested through a Q test based on analysis of variance, namely: age (years), % women, body mass index (BMI), total intervention duration (weeks), weekly frequency, session duration (min), TESTEX scale, and Journal Impact Factor. The risk of bias across studies was analyzed through funnel plots with effect sizes (*x*-axis) versus the SMD to each group of study (*y*-axis). Additionally, the nonparametric “trim and fill” method of Duval and Tweedie was also used to test and correct potential publication biases (Hoffman, 2019).

## Results

### Study Selection and Quality Assessment

**Figure 1.**
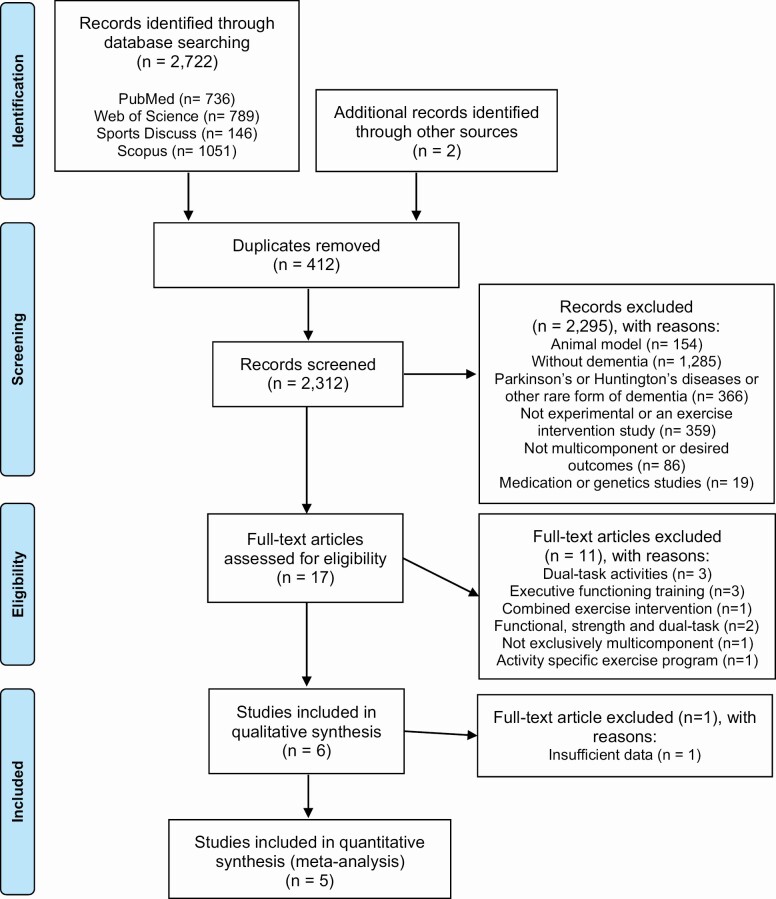
Preferred Reporting Items for Systematic Reviews and Meta-Analyses (PRISMA) flow chart diagram showing the articles screened and included in the meta-analysis study (*n* = 5).

A total of 2,312 records were retained after removing duplicates. Of these, 2,295 trials were excluded based on titles and abstracts. The reasons were samples not composed of human individuals with dementia (*k* = 1,805); interventions without exercise (medication, genetics, behavioral strategies, etc.), multimodal (*k* = 414), or trials that did not apply MT or did not report the presently investigated outcomes (*k* = 86). Of the 17 manuscripts retained for full-text review, only five satisfied all inclusion and exclusion criteria and were included in the meta-analysis. For the qualitative analysis, however, six studies were considered. Figure 1 presents the *PRISMA* literature review flowchart.

### Sample and Study Characteristics

Six controlled trials (Barreto et al., 2017; Bürge et al., 2017; Rolland et al., 2007; Sampaio et al., 2019; Steinberg et al., 2009; Vreugdenhil et al., 2012) were included in qualitative analysis. Of these, one was a nonrandomized trial (Sampaio et al., 2019) and two were pilot studies (Barreto et al., 2017; Steinberg et al., 2009). As presented in Table 1, sample sizes across the studies ranged from 27 (Steinberg et al., 2009) to 160 (Bürge et al., 2017) participants clinically diagnosed with dementia (*n* = 489), with a mean age of 80.9 years (age range: 51–93), 67.48% (*n* = 330) were women and presented an MMSE mean score of 15.03 ± 5.69 points, or 81% of participants had a total score less than 20 points.

**Table 1.** Summary of the Included Studies Characteristics

Study; country	Study design	Sample characteristics				Primary and secondary outcomes			Control group
		*N* ^a^ % Female; mean (*SD* or range)	Type of dementia	Clinical dementia criteria	Stage of dementia (MMSE or CDR score)^b^	Physical fitness	ADL functionality	Cognitive function	
Barreto et al. (2017); France	Pilot RCT	97 IG: 93.2%; 88.3 (5.1) CG: 76.6%; 86.9 (5.8)	AD, VaD, MD	DSM-IV	MMSE ≤20 IG: 11.4 (6.2) CG: 10.8 (5.5)	Time vs. Group baseline and 6 months: SPPB (*p* = .22) Gait speed (*p* = .30)	IG vs. CG 3 and 6 months, respectively: ADCS-ADL-sev (*p* < .001) ADCS-ADL-sev (*p* = .23) Time vs. Group baseline and 6 months: ADCS-ADL-sev (*p* = .19)	Time vs. Group baseline and 6 months: MMSE (*p* = .43)	Social activity group (2× week, 60 min, 24 weeks)
Bürge et al. (2017); Switzerland and Belgium	Multisite RCT	170 IG: 48.7%; 81.7 (7.7) CG: 53.7%; 81.1 (7.7)	AD, VaD, MD, LbD, FtD, SD	ICD-10	MMSE ≤20 IG: 84.2% CG: 78.1% CDR = 3 IG: 32.1% CG: 34.1%	N/A	Time vs. Group baseline and 2 weeks follow-up: BI (*p* = .001) FIM (*p* = .002)	N/A	Social group (videos or other activities)
Rolland et al. (2007); France	RCT	134 IG: 71.7%; 82.8 (7.8) CG: 79.1%; 83.1 (7.0)	AD	NINCDS-ADRDA	MMSE ≤25 IG: 9.7(6.8) CG: 7.9(6.4)	IG vs. CG baseline and 6 months: Walking Speed Test (*p* = .01) Get Up and Go Test (*p* = .68) One-leg Balance Test (*p* = .47) IG vs. CG after 12 months: Walking Speed Test (*p* = .002) Get Up and Go Test (*p* = .31) One-leg Balance Test (*p* = .34)	IG vs. CG baseline and 6 months: KATZ-ADL (*p* = .51) IG vs. CG after 12 months: KATZ-ADL (*p* = .02)	N/A	Routine medical care
Sampaio et al. (2019); Portugal	Non-RCT	37 IG: 78.9%; 84.8 (5.9) CG: 72.2%; 83.3 (5.3)	AD	Not reported	MMSE IG: 14.9 (6.0) CG: 16.1 (4.2) CDR IG: 1.68 (0.48) CG: 1.67 (0.49)	Time vs. Group baseline and 6 months: Chair Stand Test (*p* < .001) Arm Curl Test (*p* < .001) Chair Sit-and-Reach Test (*p* < .001) Back Scratch Test (*p* = .022) 8-Foot Up and Go Test (*p* = .029) 2-min Step Test (*p* < .001)	N/A	Time vs. Group baseline and 6 months: MMSE (*p* = .008)	Routine medical care
Steinberg et al. (2009); United States	Pilot RCT	27 IG: 71.4%; 76.5 (3.9) CG: 69.2%; 74.0 (8.1)	AD	NINCDS-ADRDA	MMSE IG: 20.1 (5.1) CG: 15.5 (5.4)	Time vs. Group baseline and 12 weeks: Jebsen Total Time Test (*p* = .04) Walking Speed Test (*p* = .77) 5 Chair Sit-to-Stand Test (*p* = .22)	N/A	Time vs. Group baseline and 12 weeks: BNT (*p* = .26) HVLT (*p* = .19)	Home safety assessment
Vreugdenhil et al. (2012); Australia	RCT	40 IG: 45%; 73.5 (51–83) CG: 75%; 74.7 (58–89)	UD, AD	DSM-IV NINCDS-ADRDA	MMSE IG: 22.9 (5.0) CG: 21.0 (6.3)	Time vs. Group baseline and 4 months: Functional Reach (*p* = .032) TUG (*p* = .004) Sit-to-Stand Test (*p* < .001)	Time vs. Group baseline and 4 months: BI (*p* = .047) IADL (*p* = .007)	Time vs. Group baseline and 4 months: MMSE (*p* = .001) ADAS-Cog (*p* = .001)	Usual treatment

*Notes:* AD = Alzheimer’s disease; ADAS-Cog = Alzheimer’s Disease Cognitive Scale - Cognitive Subscale; ADCS-ADL-sev = Alzheimer’s Disease Cooperative Study Activities of Daily Living Inventory for Severe Alzheimer’s Disease; BI = Barthel Index of Activities of Daily Living; BNT = Boston Naming Test; CDR = Clinical Dementia Rating; CG = control group; DSM-IV = Diagnostic and Statistical Manual of Mental Disorders 4th Edition; FIM = Functional Independence Measure; FtD = frontotemporal dementia; HVLT = Hopkins Verbal Learning Test; IADL = instrumental activities of daily living; ICD-10 = International Classification of Diseases 10th Revision; IG = intervention group; KATZ-ADL = Katz Index of ADLs; LbD = Lewy body dementia; MD = mixed dementia; MMSE = Mini-Mental State Examination; N/A = not applicable; NINCDS-ADRDA = National Institute of Neurological and Communicative Disorders and Stroke–Alzheimer’s Disease and Related Disorders Association; RCT = randomized controlled trial; SD = subcortical dementia; SFT = Senior Fitness Test; SPPB = short physical performance battery; TUG = Timed Up and Go Test; UD = undefined dementia; VaD = vascular disease.

^a^Sample size at baseline.

^b^Mean (*SD*) or percentage.

### Dropouts, Adherence, and Adverse Events

The greatest attrition rate occurred in the study conducted by Rolland et al. (2007), specifically among controls (19%). Sampaio et al. (2019) reported an attrition rate of 18.9%—seven of 37 participants dropped out in both groups from initial to final assessments. Barreto et al. (2017) reported a dropout rate of 6.2% and 12.4% after 3 and 6 months of intervention, respectively. In Bürge et al.’s (2017) study, 10 of 170 participants failed to finish the MT intervention, representing an attrition rate of 5.88%. Vreugdenhil et al. (2012) evaluated 27 participants at baseline, all of whom completed the final evaluations. Steinberg et al. (2009) did not report information on the attrition rate.

Regarding the mean adherence to MT interventions, three studies reported rates around 70%–80% (Barreto et al., 2017; Sampaio et al., 2019; Steinberg et al., 2009). In the study conducted by Bürge et al. (2017), participants in experimental and control groups were present in 13 of 20 sessions. Similarly, in Rolland et al.’s (2007) study, most participants had low adherence (participated in less than 30 sessions). Vreugdenhil et al. (2012) did not report the adherence levels to their MT intervention.

Three studies addressed potential adverse events of MT interventions for older adults with dementia (Barreto et al., 2017; Rolland et al., 2007; Steinberg et al., 2009), including mortality, hospitalizations, fractures, falls, and other nonserious and serious events. Barreto et al. (2017) found a greater occurrence of falls in the control group, and Rolland et al. (2007) reported higher hospitalization rates among patients who exercised. Although Steinberg et al. (2009) reported serious adverse events in individuals assigned to the intervention group, none of them was due to the MT intervention.

### Design of the Interventions

Routine medical care (Rolland et al., 2007; Sampaio et al., 2019; Vreugdenhil et al., 2012) or other specific health-related care (Steinberg et al., 2009) was the most frequent intervention offered to control groups. Two studies offered social activities for the control groups. Barreto et al. (2017) proposed 1-h sessions of arts and crafts or therapeutic music, twice a week for 24 weeks. In Bürge et al.’s (2017) study, patients watched videos or played social games 5 times a week for 30 min.

The length of exercise interventions (described in Table 2) was between 4 weeks and 12 months, twice a week (Barreto et al., 2017; Rolland et al., 2007; Sampaio et al., 2019), 5 times a week (Bürge et al., 2017), or on a daily basis (Steinberg et al., 2009; Vreugdenhil et al., 2012). The duration of exercise sessions ranged from 30 to 60 min (51.3 ± 12.4 min). Vreugdenhil et al.’s (2012) intervention consisted of 10 simple exercises in addition to at least 30 min of brisk walking. Participants in Steinberg et al.’s (2009) study were instructed to achieve daily specific scores in three physical components. Older adults with AD accrued points for each activity totally or partially completed (as registered on diaries by their cares), considering the predefined weekly goals for aerobics (six points) and strength/balance (four points each). These were the only studies where caregivers were included in the implementation of the exercise interventions (Steinberg et al., 2009; Vreugdenhil et al., 2012).

**Table 2.** Multicomponent Exercise Programs Description

Study	Intensity (minutes/session)	Frequency (sessions/week)	Duration	Exercise protocol	Exercise intensity	Design setting	Supervision
Barreto et al. (2017)	60	2	24 weeks, 48 sessions	Warm-up 10 min Coordination/balance 10 min Strength 10–15 min Aerobics 20 min Cool-down 5–10 min	Moderate^a^	Seven nursing homes	Exercise instructors
Bürge et al. (2017)	30	5	4 weeks, 20 sessions	Rolland et al.’s (2007) exercise protocol: strength, flexibility, walking, and balance training	Moderate^b^	Five acute psychiatric settings	Occupational, physical or psychomotor therapists
Rolland et al. (2007)	60	2	12 months, 88 sessions	Inside circular walking trail created and adapted to each exercise group After warm-up and stretching, individuals were motivated to walk (at least during half of the session) Strength, flexibility, and balance exercises intercepted on predetermined stations along the trail	Moderate^b^	Five nursing homes	Occupational therapist
Sampaio et al. (2019)	45–55	2	6 months, 48 sessions	Warm-up 5–10 min Specific training (Aerobics 15 min and Strength, Coordination/Balance 15–20 min) Cool-down (flexibility and respiratory exercises) 5 min	Moderate^c^	Nine nursing homes	Exercise instructors
Steinberg et al. (2009)	Points for each aerobic, strength, and balance completed activities Weekly goals: 6 aerobic points, 4 strength and balance points	Daily, reported on weekly diaries	12 weeks	Aerobics (brisk walking) Strength training (with resistive bands and ankle weights) Balance and flexibility training (tandem walks, chair sit to stands, forward and backward walks, change of gravity center)	Moderate^b^	Home-based exercise program	Caregivers and exercise physiologist
Vreugdenhil et al. (2012)	At least 30 min of brisk walking and 10 simple exercises	Daily	4 months	Home Support Exercise Program: 10 simple exercises for upper and lower body strength, balance training; and at least 30 min of endurance (brisk walking)	Not monitored^d^	Home-based exercise program	Caregivers

^a^Any objective measure used.

^b^Contacted by email for details, but no response received.

^c^Borg Scale of Perceived Exertion.

^d^Quote: “increased difficulty on exercises over time”; exercise intensity was not objectively monitored.

Considering the size of exercise groups, Barreto et al. (2017) did not report any information. According to Rolland et al. (2007), each group was formed of two to seven participants (mean 5.2), based on their baseline physical abilities, cognitive function, behavior disturbances, and affinity. Bürge et al. (2017) formed groups of at most four participants, and in Sampaio et al.’s (2019) study each exercise group consisted of four to seven individuals with dementia.

In three studies, music accompanied the exercise sessions (Bürge et al., 2017; Rolland et al., 2007; Sampaio et al., 2019).

### Major Outcomes

**Figure 2.**
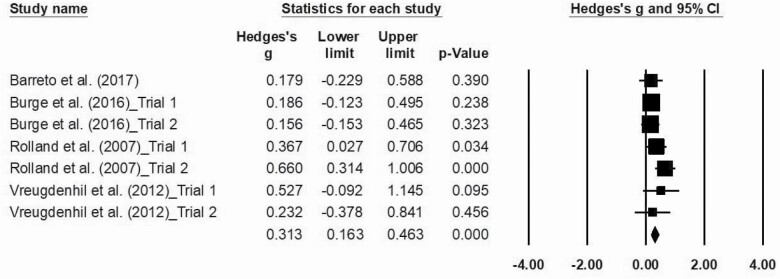
Effect of multicomponent training exercise interventions on activities of daily living’s functionality.

**Figure 3.**
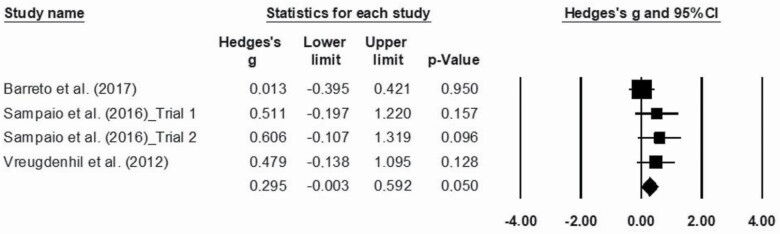
Effect of multicomponent training exercise interventions on cognition.

**Figure 4.**
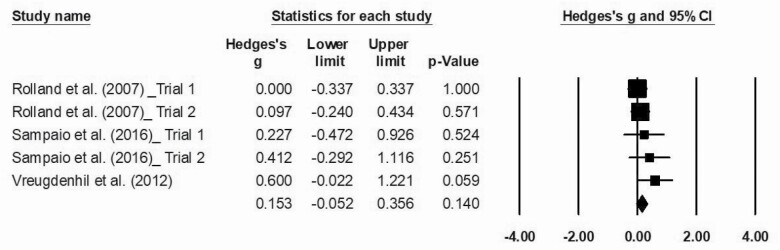
Effect of multicomponent training exercise interventions on agility.

The statistical analysis was conducted with 16 trials, identified through the five studies included in the meta-analysis. Meta-analysis was performed regarding ADL functionality, cognition, and agility, as data were not sufficient to proceed to further analysis regarding other physical fitness components. The effects sizes of MT are represented in Figures 2–4, respectively. Overall, this analysis revealed a positive impact of MT exercise on ADL functionality with an ES of 0.313 (0.16–0.46; *p* < .01). Regarding cognition (ES = 0.29 [−0.00 to 0.59]; *p* = .05) and agility (ES = 0.153 [−0.052 to 0.356]; *p* = .14), results showed that MT intervention did not promote modification on these outcomes. The *I*^2^ statistic revealed low heterogeneity for the studies that investigated ADL functionality (*I*^2^ = 8.1; *p* < .01), cognition (*I*^2^ = 7.8; *p* = .35), and agility (*I*^2^ = 0.0; *p* = .48).

### Subgroup Analyses

Table 3 details the moderators of ADL effect, with the purpose of addressing the quantitative influence of characteristics of the included studies or their participants on the effect size of ADL functionality. Thus, only the total time (in weeks) of the MT program duration influenced the ADL effect. For each additional week of intervention, the total effect size related to ADL functionality increased by 0.007 (*p <* .05). Neither age of participants (*p* = .73), gender (*p* = .47), or BMI (*p* = .94) nor other aspects concerning the studies’ quality (*p* = .75) demonstrated differences in intervention effects.

**Table 3.** Meta-Regression Analyses of MT Intervention Characteristics Regarding ADL Functionality

Variable	*N* (trials)	SLOPE	*p* Value
Age (years)	7	−0.006	.73
% women	7	0.003	.47
BMI	5	0.015	.94
Total intervention duration (weeks)	7	0.007	.04*
Weekly frequency	7	−0.044	.27
Session duration (min)	7	0.008	.11
TESTEX scale	7	−0.024	.75
Journal Impact Factor	7	0.007	.24

*Note:* ADL = activities of daily living; BMI = body mass index; MT = multicomponent training.

*Statistically significant difference (*p* < .05).

### Risk of Bias

**Figure 5.**
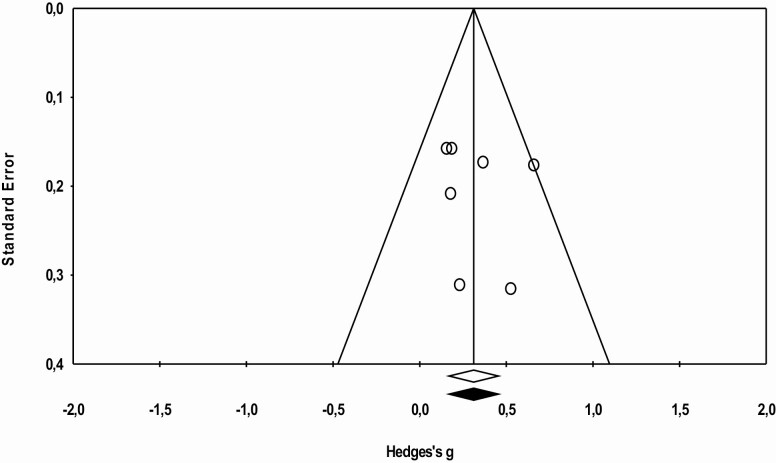
Funnel plot of standard error by Hedges’ *g* (activities of daily living’s functionality).

**Figure 6.**
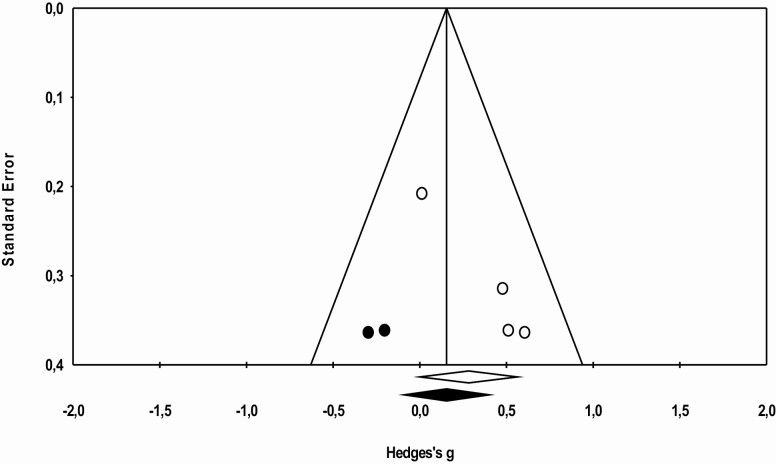
Funnel plot of standard error by Hedges’ *g* (cognition).

**Figure 7.**
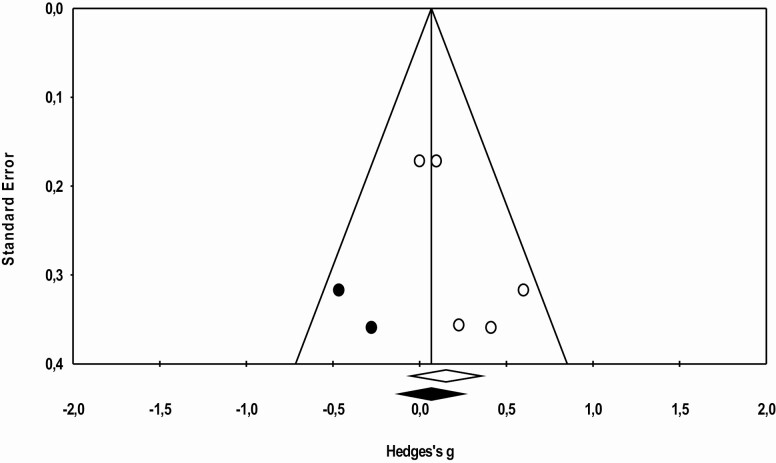
Funnel plot of standard error by Hedges’ *g* (agility).

Publication bias effect was verified using the Egger test and analyzed through funnel plots combined with Duval and Tweedie’s “trim and fill” correction method (Figures 5–7). No publication bias was identified for the analyzed variables (Figure 5. ADL: *g* observed = 0.311 [0.169–0.454], *g* adjusted = 0.311 [0.169–0.454]; Figure 6. Cognition: *g* observed = 0.281 [−0.000–0.563], *g* adjusted = 0.154 [−0.090–0.400]; and Figure 7. Agility: *g* observed = 0.152 [−0.050 to 0.355], *g* adjusted = 0.067 [−0.118 to 0.253]).

### Sensitivity Analyses

**Figure 8.**
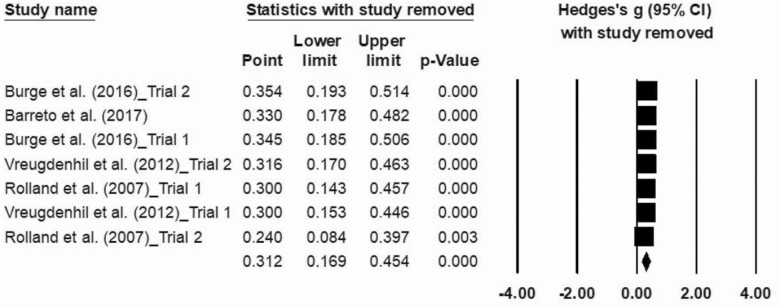
Sensitivity analysis of activities of daily living’s effect size.

The sensitivity analyses with study removal over the effects sizes on ADL trials (Figure 8) did not show any influence on obtained results.

## Discussion

### Summary of Evidence

This review included five articles with a total of 438 participants. A sixth article, referring to Steinberg et al.’s (2009) study, was initially included, but due to insufficient data, it could not be considered in the statistical analysis. Most participants were women, with an average age older than 73 years. AD was the most referred type of dementia, ranging from light to severe stages.

High heterogeneity was found among intervention design, evaluation instruments, and measures, as well as on the description of dementia diagnosis and/or stage. The MT intervention programs varied widely regarding the following aspects: a combination of the different physical fitness components and the amount of time dedicated to each of them; frequency, duration, and intensity of exercise sessions; settings where MT intervention was implemented (e.g., nursing homes, psychiatric hospitals, or patients’ homes). Such diversity was also observed across physical fitness levels, functional abilities, and dementia stages, as well as regarding the sizes of exercise groups and professionals’ background involved in prescription, supervision, and evaluation. Therefore, results must be analyzed with caution because these differences may have a significant impact on MT intervention results (Forbes et al., 2015; Livingston et al., 2017). Regarding control groups, routine medical care or other specific health-related care was the most frequent intervention offered to older adults diagnosed with dementia. As highlighted in previous research (Borges-Machado et al., 2019; van der Wardt et al., 2017), studies designed for individuals diagnosed with dementia should consider other types of interventions, such as monthly recreational sessions, due to retention purposes and accurate ethical procedures.

Significantly high attrition rates were reported (nearly 20%); however, adherence to the intervention was also relatively satisfactory—nearly 70%–80% of attendance levels on three of the included studies. These differences between attrition and adherence rates must be analyzed with caution, considering the possible influence of design settings, and the inclusion of family caregivers. Thereby, the studies conducted at home-based settings did not report any dropouts, possibly due to caregivers’ influence on promoting physical exercise (Almeida et al., 2019; Steinberg et al., 2009; Vreugdenhil et al., 2012). In contrast, the MT intervention conducted with hospitalized older adults with dementia in acute psychiatric settings revealed a low overall adherence rate to the program, whose reasons have been appropriately presented (Bürge et al., 2017). No serious events attributed to the studies were reported.

This meta-analysis revealed a positive impact of MT interventions on ADL functionality. Concerning cognitive function and physical fitness, specifically agility, results showed that MT intervention did not influence these outcomes.

In the analysis of ADL moderators, only the MT intervention duration influenced the effect size of this outcome. Heyn et al. (2004) also reinforced that longer exercise interventions (more than 23 weeks) were associated with greater benefits for individuals with dementia. Exercise session frequency or duration (min) did not influence the ADL performance effect size. As stated by Forbes et al. (2015), the mitigation of dependence in ADL functionality, as a result of dementia progressing, is critical for enhancing the quality of life of both older adults with dementia and their caregivers and may prevent or delay institutionalization.

Concerning cognitive function, the MMSE (Folstein et al., 1975) was the only instrument used to measure this outcome. Although this cognitive screening tool is a worldwide reference, there is growing evidence suggesting it may be unsuitable to measure modifications on cognition of older adults diagnosed with dementia (Santana et al., 2016). Therefore, the authors may consider its use as a limitation on analyzing MT intervention effectiveness. Farina et al.’s (2014) systematic review, whose aim was to assess the effectiveness of exercise in cognitive decline within AD, also reinforced that more important than analyzing general cognitive functions, subdomain changes must be addressed through a more comprehensive cognitive test battery. Future exercise studies might consider the importance of standardization outcome measures to evaluate the cognitive function of older adults with dementia and, more importantly, include more comprehensive instruments. The high heterogeneity of study designs also contributes to the uncertainty of the effectiveness of exercise on improving cognitive function or delaying dementia progression (Erickson et al., 2019). The overall effect of MT interventions on cognition may also have been influenced by several clinical factors, such as different diagnostic medical criteria (DSM-IV, ICD-10, or NINCDS-ADRDA); variety of types of dementia; and dementia participants’ disease severity, which ranged from mild to severe stages.

Finally, we observed that MT interventions were not effective in improving agility in individuals diagnosed with dementia. Nevertheless, when analyzing the Hedges’ *g* forest plots, it is possible to verify that results favor exercise. The limited number of studies included in this analysis may explain the absence of a statistically significant effect size over this outcome. In fact, although motor function, gait speed, cardiorespiratory capacity, strength, balance, and flexibility physical components were measured, due to insufficient data, these analyses could not be performed.

To the best of our knowledge, physical fitness is not commonly considered as a primary outcome in these specific trials. However, considering the decline of increased physical fitness’ in older adults with dementia, and its impact on their ability to independently perform ADL functionality without fatigue, it seems imperative to be considered as such. Therefore, future exercise studies for people with dementia need to focus on measuring physical fitness effects through reliable instruments, minimizing the effects of cognitive impairment on physical performance (Forbes et al., 2015), as previously validated or, at least, tested by several authors (Blankevoort et al., 2013; Burton et al., 2015; Gonçalves et al., 2018; Lamb & Keene, 2017; Tappen et al., 1997).

Overall, this review and meta-analytic study highlight the impact of MT interventions (i.e., comprising several components of physical fitness) on promoting/mitigating the decline of dementia participants’ independence on performing daily tasks, which are critical to enhance theirs’ and caregivers’ quality of life. However, there is no strong evidence concerning the positive effects of MT training methodology on specific cognitive abilities and physical fitness. Further studies are needed to acknowledge these benefits.

As long as review and meta-analytic studies only focus on generalized effects of exercise on dementia, particularly on cognitive outcomes, aerobic exercise at moderate intensity for older adults with dementia at mild to moderate stages of the disease will remain the single existing guideline on exercise prescription for this health condition (American College of Sports Medicine, 2018; Erickson et al., 2019; Groot et al., 2016; Skinner et al., 2018; Smith et al., 2010). Therefore, research studies must identify the triad: stage/type of dementia, FITT variables, and target outcome and defined pathway in order to effectively plan and prescribe exercise interventions for older adults with dementia (Forbes et al., 2015; Skinner et al., 2018). It is imperative that future review studies focus on specific training modalities (e.g., MT) to acknowledge its benefits upon a specific outcome, considering the different types/stages of dementia.

### Limitations

The inclusion of only five studies may be a limitation of this meta-analysis. First, the use of Baker et al.’s (2007) definition of MT methodology excludes exercise interventions that offer two or three physical fitness components, but separately on different sessions (vs. in the same session), which decreased the number of included studies. Second, the exclusion of several dementia types has also contributed to the removal of a considerable number of studies from our sample. However, these conditions imply adapted programs to patients’ mobility/functional limitations and disease progression specificities. The inclusion of only English studies may have also restricted our research.

Finally, the lack of statistical data from the included studies was also a limitation when analyzing the moderator effect of other variables on ADL functionality. Moreover, quality analysis of this review may be compromised by lack of information on several criteria, for example, on methods used to control exercise session intensity and adverse events—determinant factors considering exercise prescription for older adults diagnosed with dementia. Therefore, researchers must ensure high methodological quality trials and provide all the information necessary for study quality/reporting analysis (Smart et al., 2015), and describe statistical data, in order to guide strong recommendations of MT interventions for individuals diagnosed with dementia.

### Future Practical Implications

With the increasing number of individuals diagnosed with dementia, this syndrome is now considered one of the main age-related health problems affecting modern society (Alzheimer’s Association, 2020; Arvanitakis et al., 2019), demanding a conjoint action of social and health care services to create and implement effective nonpharmacological strategies to treat and care for the diagnosed individuals and respective caregivers (WHO, 2019). Future research studies must consider MT intervention as a potentially effective training methodology on decreasing the progression of ADL dependence on older adults with dementia, which may affect the caregivers’ ability to sustain their role. Exercise programs should be conducted by qualified professionals, in small size groups, and including enjoyable and appropriate activities for the participants, in order to sustain physical activity over a long period. Well-designed clinical trials should be conducted at community-based settings and preferably should include caregivers (Forbes et al., 2015; Heisz et al., 2016).

## Conclusions

This meta-analysis shows that MT interventions are effective in improving ADL performance of individuals diagnosed with dementia. Findings suggest that the program duration (long-term interventions) had a superior influence on daily functionality than exercise session frequency and duration. Despite the methodological limitations, high-quality assumptions attested by heterogeneity, risk of bias, and sensitivity analysis results were achieved. Finally, our results reinforce the need for future randomized controlled trials to acknowledge the effectiveness of MT interventions on cognitive function and physical fitness of older adults diagnosed with dementia, considering the combination of different intensity/frequency/time variables for different types/stages of dementia.

## Supplementary Material

gnaa091_suppl_Supplementary_Material

## References

[CIT0001] Almeida, S I L d, Gomes da Silva, M, & Marques, A S P d D. (2019). Home-based physical activity programs for people with dementia: Systematic review and meta-analysis. The Gerontologist. Advance online publication. doi:10.1093/geront/gnz176 31858111

[CIT0002] Alty, J, Farrow, M, & Lawler, K. (2020). Exercise and dementia prevention. Practical Neurology, 0, 1–7. doi:10.1136/practneurol-2019-002335 31964800

[CIT0003] Alzheimer’s Association . (2020). 2020 Alzheimer’s disease facts and figures. Alzheimer’s & Dementia, 16(3), 391–460. doi:10.1002/alz.12068

[CIT0004] Alzheimer’s Disease International . (2018). World Alzheimer Report 2018—The state of the art of dementia research: New frontiers. Author.

[CIT0005] American College of Sports Medicine . (2017). ACSM’s guidelines for exercise testing and prescription (10th ed.). Wolters Kluwer. 10.1249/JSR.0b013e31829a68cf 23851406

[CIT0006] American College of Sports Medicine . (2018). Being active with Alzheimer’s disease. Exercise is medicine. https://www.exerciseismedicine.org/support_page.php/rx-for-health-series/. Accessed October 27, 2019.

[CIT0007] American Psychiatric Association . (2013). Neurocognitive disorders. In DSM-5: Diagnostic and statistical manual of mental disorders (Vol. 5). American Psychiatric Publishing.

[CIT0008] Arvanitakis, Z, Shah, R C, & Bennett, D A. (2019). Diagnosis and management of dementia: Review. JAMA, 322(16), 1589–1599. doi:10.1001/jama.2019.4782 PMC7462122 31638686

[CIT0009] Baker, M K, Atlantis, E, & Fiatarone Singh, M A. (2007). Multi-modal exercise programs for older adults. Age and Ageing, 36(4), 375–381. doi:10.1093/ageing/afm054 17537741

[CIT0010] Barreto, P d S, Cesari, M, Denormandie, P, Armaingaud, D, Vellas, B, & Rolland, Y. (2017). Exercise or social intervention for nursing home residents with dementia: A pilot randomized controlled trial. Journal of the American Geriatrics Society, 65(9), E123–E129. doi:10.1111/jgs.14947 28542742

[CIT0011] Becker, B J . (1988). Synthesizing standardized mean-change measures. British Journal of Mathematical and Statistical Psychology, 41, 257–278. doi:10.1111/j.2044-8317.1988.tb00901.x

[CIT0012] Blankevoort, C G, van Heuvelen, M J, Boersma, F, Luning, H, de Jong, J, & Scherder, E J. (2010). Review of effects of physical activity on strength, balance, mobility and ADL performance in elderly subjects with dementia. Dementia and Geriatric Cognitive Disorders, 30(5), 392–402. doi:10.1159/000321357 20980758

[CIT0013] Blankevoort, C G, van Heuvelen, M J, & Scherder, E J. (2013). Reliability of six physical performance tests in older people with dementia. Physical Therapy, 93(1), 69–78. doi:10.2522/ptj.20110164 22976448

[CIT0014] Blondell, S J, Hammersley-Mather, R, & Veerman, J L. (2014). Does physical activity prevent cognitive decline and dementia? A systematic review and meta-analysis of longitudinal studies. BMC Public Health, 14, 510. doi:10.1186/1471-2458-14-510 24885250 PMC4064273

[CIT0015] Borenstein, M, Hedges, L V, & Higgins, J P T. (2009). Introduction to meta-analysis. John Wiley & Sons, Ltd.

[CIT0016] Borges-Machado, F, Ribeiro, Ó, Sampaio, A, Marques-Aleixo, I, Meireles, J, & Carvalho, J. (2019). Feasibility and impact of a multicomponent exercise intervention in patients with Alzheimer’s disease: A pilot study. American Journal of Alzheimer’s Disease and Other Dementias, 34(2), 95–103. doi:10.1177/1533317518813555 PMC10852450 30525876

[CIT0017] Bürge, E, Berchtold, A, Maupetit, C, Bourquin, N, Von Gunten, A, Ducraux, D, Zumbach, S, Peeters, A, & Kuhne, N. (2017). Does physical exercise improve ADL capacities in people over 65 years with moderate or severe dementia hospitalized in an acute psychiatric setting? A multisite randomized clinical trial. International Psychogeriatrics, 29(2), 323–332. doi:10.1017/S1041610216001460 27831462

[CIT0018] Burton, E, Cavalheri, V, Adams, R, Browne, C O, Bovery-Spencer, P, Fenton, A, Campbell, B, & Hill, K. (2015). Effectiveness of exercise programs to reduce falls in older people with dementia living in the community: A systematic review and meta-analysis. Clinical Interventions in Aging, 10, 421–434. doi:10.2147/CIA.S71691 25709416 PMC4330004

[CIT0019] Card, N A . (2011). Applied meta-analysis for social science research. Guilford Publications.

[CIT0020] Carvalho, M J, Marques, E, & Mota, J. (2009). Training and detraining effects on functional fitness after a multicomponent training in older women. Gerontology, 55(1), 41–48. doi:10.1159/000140681 18562788

[CIT0021] Caspersen, C J, Powell, K E, & Christenson, G M. (1985). Physical activity, exercise, and physical fitness: Definitions and distinctions for health-related research. Public Health Reports (Washington, D.C.: 1974), 100(2), 126–131. PMC1424733 3920711

[CIT0022] Cass, S P . (2017). Alzheimer’s disease and exercise: A literature review. Current Sports Medicine Reports, 16(1), 19–22. doi:10.1249/JSR.0000000000000332 28067736

[CIT0023] Durlak, J A . (2009). How to select, calculate, and interpret effect sizes. Journal of Pediatric Psychology, 34(9), 917–928. doi:10.1093/jpepsy/jsp004 19223279

[CIT0024] Erickson, K I, Hillman, C, Stillman, C M, Ballard, R M, Bloodgood, B, Conroy, D E, Macko, R, Marquez, D X, Petruzzello, S J, & Powell, K E; FOR 2018 PHYSICAL ACTIVITY GUIDELINES ADVISORY COMMITTEE* . (2019). Physical activity, cognition, and brain outcomes: A review of the 2018 physical activity guidelines. Medicine and Science in Sports and Exercise, 51(6), 1242–1251. doi:10.1249/MSS.0000000000001936 31095081 PMC6527141

[CIT0025] Erickson, K I, Weinstein, A M, & Lopez, O L. (2012). Physical activity, brain plasticity, and Alzheimer’s disease. Archives of Medical Research, 43(8), 615–621. doi:10.1016/j.arcmed.2012.09.008 23085449 PMC3567914

[CIT0026] Farina, N, Rusted, J, & Tabet, N. (2014). The effect of exercise interventions on cognitive outcome in Alzheimer’s disease: A systematic review. International Psychogeriatrics, 26(1), 9–18. doi:10.1017/S1041610213001385 23962667

[CIT0027] Folstein, M F, Folstein, S E, & McHugh, P R. (1975). “Mini-mental state”. A practical method for grading the cognitive state of patients for the clinician. Journal of Psychiatric Research, 12(3), 189–198. doi:10.1016/0022-3956(75)90026-6 1202204

[CIT0028] Forbes, D, Forbes, S C, Blake, C M, Thiessen, E J, & Forbes, S. (2015). Exercise programs for people with dementia (review). Cochrane Database of Systematic Reviews, ( 4), CD006489. doi:10.1002/14651858.CD006489.pub4 PMC9426996 25874613

[CIT0029] Gomes-Osman, J, Cabral, D F, Morris, T P, McInerney, K, Cahalin, L P, Rundek, T, Oliveira, A, & Pascual-Leone, A. (2018). Exercise for cognitive brain health in aging: A systematic review for an evaluation of dose. Neurology. Clinical Practice, 8(3), 257–265. doi:10.1212/CPJ.0000000000000460 30105166 PMC6075983

[CIT0030] Gonçalves, A C, Cruz, J, Marques, A, Demain, S, & Samuel, D. (2018). Evaluating physical activity in dementia: A systematic review of outcomes to inform the development of a core outcome set. Age and Ageing, 47(1), 34–41. doi:10.1093/ageing/afx135 28985262

[CIT0031] Groot, C, Hooghiemstra, A M, Raijmakers, P G, van Berckel, B N, Scheltens, P, Scherder, E J, van der Flier, W M, & Ossenkoppele, R. (2016). The effect of physical activity on cognitive function in patients with dementia: A meta-analysis of randomized control trials. Ageing Research Reviews, 25, 13–23. doi:10.1016/j.arr.2015.11.005 26607411

[CIT0032] Heisz, J J, Kovacevic, A, Clark, I B, & Vandermorris, S. (2016). Evaluation of a community-based exercise program for managing Alzheimer’s disease. Journal of the American Geriatrics Society, 64(4), 884–886. doi:10.1111/jgs.14054 27100587

[CIT0033] Hernández, S S, Sandreschi, P F, da Silva, F C, Arancibia, B A, da Silva, R, Gutierres, P J, & Andrade, A. (2015). What are the benefits of exercise for Alzheimer’s disease? A systematic review of the past 10 years. Journal of Aging and Physical Activity, 23(4), 659–668. doi:10.1123/japa.2014-0180 25414947

[CIT0034] Heyn, P, Abreu, B C, & Ottenbacher, K J. (2004). The effects of exercise training on elderly persons with cognitive impairment and dementia: A meta-analysis. Archives of Physical Medicine and Rehabilitation, 85(10), 1694–1704. doi:10.1016/j.apmr.2004.03.019 15468033

[CIT0035] Higgins, J P, Thompson, S G, Deeks, J J, & Altman, D G. (2003). Measuring inconsistency in meta-analyses. BMJ (Clinical Research ed.), 327(7414), 557–560. doi:10.1136/bmj.327.7414.557 PMC192859 12958120

[CIT0036] Hoffman, J I E . (2019). Meta-analysis. In J I E Hoffman (Ed.), Basic biostatistics for medical and biomedical practitioners (2nd ed., pp. 621–629). Academic Press.

[CIT0037] Kirk-Sanchez, N J, & McGough, E L. (2014). Physical exercise and cognitive performance in the elderly: Current perspectives. Clinical Interventions in Aging, 9, 51–62. doi:10.2147/CIA.S39506 24379659 PMC3872007

[CIT0038] Kivipelto, M, Mangialasche, F, & Ngandu, T. (2018). Lifestyle interventions to prevent cognitive impairment, dementia and Alzheimer disease. Nature Reviews. Neurology, 14(11), 653–666. doi:10.1038/s41582-018-0070-3 30291317

[CIT0039] Lam, F M, Huang, M Z, Liao, L R, Chung, R C, Kwok, T C, & Pang, M Y. (2018). Physical exercise improves strength, balance, mobility, and endurance in people with cognitive impairment and dementia: A systematic review. Journal of Physiotherapy, 64(1), 4–15. doi:10.1016/j.jphys.2017.12.001 29289581

[CIT0040] Lamb, S E, & Keene, D J. (2017). Measuring physical capacity and performance in older people. Best Practice & Research. Clinical Rheumatology, 31(2), 243–254. doi:10.1016/j.berh.2017.11.008 29224699

[CIT0041] Livingston, G, Sommerlad, A, Orgeta, V, Costafreda, S G, Huntley, J, Ames, D, Ballard, C, Banerjee, S, Burns, A, Cohen-Mansfield, J, Cooper, C, Fox, N, Gitlin, L N, Howard, R, Kales, H C, Larson, E B, Ritchie, K, Rockwood, K, Sampson, E L, ... Mukadam, N. (2017). Dementia prevention, intervention, and care. Lancet (London, England), 390(10113), 2673–2734. doi:10.1016/S0140-6736(17)31363-6 28735855

[CIT0042] Moher, D, Shamseer, L, Clarke, M, Ghersi, D, Liberati, A Petticrew, M, Shekelle, P, Stewart, L A& PRISMA-P Group. (2015). Preferred reporting items for systematic review and meta-analysis protocols (PRISMA-P) 2015 statement. Systematic Reviews, 4(1), 1. doi:10.1186/2046-4053-4-1 25554246 PMC4320440

[CIT0043] Norton, S, Matthews, F E, Barnes, D E, Yaffe, K, & Brayne, C. (2014). Potential for primary prevention of Alzheimer’s disease: An analysis of population-based data. The Lancet. Neurology, 13(8), 788–794. doi:10.1016/S1474-4422(14)70136-X 25030513

[CIT0044] Pitkälä, K, Savikko, N, Poysti, M, Strandberg, T, & Laakkonen, M L. (2013). Efficacy of physical exercise intervention on mobility and physical functioning in older people with dementia: A systematic review. Experimental Gerontology, 48(1), 85–93. doi:10.1016/j.exger.2012.08.008 22960590

[CIT0045] Quaglio, G, Brand, H, & Dario, C. (2016). Fighting dementia in Europe: the time to act is now. The Lancet. Neurology, 15(5), 452–454. doi:10.1016/S1474-4422(16)00079-X 26987700

[CIT0046] Radak, Z, Hart, N, Sarga, L, Koltai, E, Atalay, M, Ohno, H, & Boldogh, I. (2010). Exercise plays a preventive role against Alzheimer’s disease. Journal of Alzheimer’s Disease, 20(3), 777–783. doi:10.3233/JAD-2010-091531 20182027

[CIT0047] Rolland, Y, Pillard, F, Klapouszczak, A, Reynish, E, Thomas, D, Andrieu, S, Rivière, E, & Vellas, B. (2007). Exercise program for nursing home residents with Alzheimer’s disease: A 1-year randomized, controlled trial. Journal of the American Geriatrics Society, 55(2), 158–165. doi:10.1111/j.1532-5415.2007.01035.x 17302650

[CIT0048] Sallis, J F, Bull, F, Guthold, R, Heath, G W, Inoue, S, Kelly, P, Oyeyemi, A L, Perez, L G, Richards, J, & Hallal, P C. (2016). Physical activity 2016: Progress and challenges. Progress in Physical Activity Over the Olympic Quadrennium, 388(10051), 1325–1336. doi:10.1016/S0140-6736(16)30581-5 27475270

[CIT0049] Sampaio, A, Marques, E A, Mota, J, & Carvalho, J. (2019). Effects of a multicomponent exercise program in institutionalized elders with Alzheimer’s disease. Dementia (London, England), 18(2), 417–431. doi:10.1177/1471301216674558 27756836

[CIT0050] Sampaio, A, Marques-Aleixo, I, Seabra, A, Mota, J, Marques, E, & Carvalho, J. (2020). Physical fitness in institutionalized older adults with dementia: Association with cognition, functional capacity and quality of life. Aging Clinical and Experimental Research. doi:10.1007/s40520-019-01445-7 PMC7591410 31927709

[CIT0051] Santana, I, Duro, D, Lemos, R, Costa, V, Pereira, M, Simoes, M R, & Freitas, S. (2016). Mini-Mental State Examination: Screening and diagnosis of cognitive decline, using new normative data. Acta Medica Portuguesa, 29(4), 240–248. doi:10.20344/amp.6889 27349775

[CIT0052] Skinner, S N, Ellis, M P, & Pa, J. (2018). The effects of physical activity on cognition, dementia risk, and brain health. In G E Smith & S T Farias (Eds.), APA handbook of dementia (pp. 381–398). American Psychological Association.

[CIT0053] Smart, N A, Waldron, M, Ismail, H, Giallauria, F, Vigorito, C, Cornelissen, V, & Dieberg, G. (2015). Validation of a new tool for the assessment of study quality and reporting in exercise training studies: TESTEX. International Journal of Evidence-Based Healthcare, 13(1), 9–18. doi:10.1097/XEB.0000000000000020 25734864

[CIT0054] Smith, P J, Blumenthal, J A, Hoffman, B M, Cooper, H, Strauman, T A, Welsh-Bohmer, K, Browndyke, J N, & Sherwood, A. (2010). Aerobic exercise and neurocognitive performance: A meta-analytic review of randomized controlled trials. Psychosomatic Medicine, 72(3), 239–252. doi:10.1097/PSY.0b013e3181d14633 20223924 PMC2897704

[CIT0055] Steinberg, M, Leoutsakos, J M, Podewils, L J, & Lyketsos, C G. (2009). Evaluation of a home-based exercise program in the treatment of Alzheimer’s disease: The Maximizing Independence in Dementia (MIND) study. International Journal of Geriatric Psychiatry, 24(7), 680–685. doi:10.1002/gps.2175 19089875 PMC5172460

[CIT0056] Tappen, R M, Roach, K E, Buchner, D, Barry, C, & Edelstein, J. (1997). Reliability of physical performance measures in nursing home residents with Alzheimer’s disease. The Journals of Gerontology, Series A: Biological Sciences and Medical Sciences, 52(1), 52–55. doi:10.1093/gerona/52a.1.m52 PMC1965283 9008669

[CIT0057] Toraman, N F, Erman, A, & Agyar, E. (2004). Effects of multicomponent training on functional fitness in older adults. Journal of Aging and Physical Activity, 12(4), 538–553. doi:10.1123/japa.12.4.538 15851825

[CIT0058] van der Wardt, V, Hancox, J, Gondek, D, Logan, P, Nair, R D, Pollock, K, & Harwood, R. (2017). Adherence support strategies for exercise interventions in people with mild cognitive impairment and dementia: A systematic review. Preventive Medicine Reports, 7, 38–45. doi:10.1016/j.pmedr.2017.05.007 28593121 PMC5447393

[CIT0059] Vreugdenhil, A, Cannell, J, Davies, A, & Razay, G. (2012). A community-based exercise programme to improve functional ability in people with Alzheimer’s disease: A randomized controlled trial. Scandinavian Journal of Caring Sciences, 26(1), 12–19. doi:10.1111/j.1471-6712.2011.00895.x 21564154

[CIT0060] Winblad, B, Amouyel, P, Andrieu, S, Ballard, C, Brayne, C, Brodaty, H, Cedazo-Minguez, A, Dubois, B, Edvardsson, D, Feldman, H, Fratiglioni, L, Frisoni, G B, Gauthier, S, Georges, J, Graff, C, Iqdal, K, Jessen, F, Johansson, G, Jonsson, L, ... Zetterberg, H. (2016). Defeating Alzheimer’s disease and other dementias: A priority for European science and society. The Lancet Neurology, 15(5), 455–532. doi:10.1016/S1474-4422(16)00062-4 26987701

[CIT0061] World Health Organization . (2017). Global action plan on the public health response to dementia: 2017–2025. Author.

[CIT0062] World Health Organization . (2018). Towards a dementia plan: A WHO guide. Author.

[CIT0063] World Health Organization . (2019). Risk reduction of cognitive decline and dementia. Author. 31219687

