# Supplementary material for: Effectiveness of Multicomponent Exercise Interventions in Older Adults With Dementia: A Meta-Analysis
Source: Gerontologist. 2020 Jul 11;61(8):e449–62. doi: 10.1093/geront/gnaa091 (PMC8599205; doi:10.1093/geront/gnaa091)
Supplement: gnaa091_suppl_Supplementary_Material [file gnaa091_suppl_supplementary_material.docx]

**Online Supplementary Material**

**Supplementary Table 1.** PRISMA Checklist

| **Section/topic** | **#** | **Checklist item** | **Reported on page** |
| --- | --- | --- | --- |
| **TITLE** | | |  |
| Title | 1 | Identify the report as a systematic review, meta-analysis, or both. | 1 |
| **ABSTRACT** | | |  |
| Structured summary | 2 | Provide a structured summary including, as applicable: background; objectives; data sources; study eligibility criteria, participants, and interventions; study appraisal and synthesis methods; results; limitations; conclusions and implications of key findings; systematic review registration number. | 3-4 |
| **INTRODUCTION** | | |  |
| Rationale | 3 | Describe the rationale for the review in the context of what is already known. | 4-7 |
| Objectives | 4 | Provide an explicit statement of questions being addressed with reference to participants, interventions, comparisons, outcomes, and study design (PICOS). | 7 |
| **METHODS** | | |  |
| Protocol and registration | 5 | Indicate if a review protocol exists, if and where it can be accessed (e.g., Web address), and, if available, provide registration information including registration number. | 8 |
| Eligibility criteria | 6 | Specify study characteristics (e.g., PICOS, length of follow-up) and report characteristics (e.g., years considered, language, publication status) used as criteria for eligibility, giving rationale. | 9 |
| Information sources | 7 | Describe all information sources (e.g., databases with dates of coverage, contact with study authors to identify additional studies) in the search and date last searched. | 8-9 |
| Search | 8 | Present full electronic search strategy for at least one database, including any limits used, such that it could be repeated. | 9, and Supplementary Table 2 |
| Study selection | 9 | State the process for selecting studies (i.e., screening, eligibility, included in systematic review, and, if applicable, included in the meta-analysis). | 9 |
| Data collection process | 10 | Describe method of data extraction from reports (e.g., piloted forms, independently, in duplicate) and any processes for obtaining and confirming data from investigators. | 9-10 |
| Data items | 11 | List and define all variables for which data were sought (e.g., PICOS, funding sources) and any assumptions and simplifications made. | 9-10 |
| Risk of bias in individual studies | 12 | Describe methods used for assessing risk of bias of individual studies (including specification of whether this was done at the study or outcome level), and how this information is to be used in any data synthesis. | 10 |
| Summary measures | 13 | State the principal summary measures (e.g., risk ratio, difference in means). | 10-11 |
| Synthesis of results | 14 | Describe the methods of handling data and combining results of studies, if done, including measures of consistency (e.g., I^2^) for each meta-analysis. | 11-12 |
| Risk of bias across studies | 15 | Specify any assessment of risk of bias that may affect the cumulative evidence (e.g., publication bias, selective reporting within studies). | 12 |
| Additional analyses | 16 | Describe methods of additional analyses (e.g., sensitivity or subgroup analyses, meta-regression), if done, indicating which were pre-specified. | 11 |
| **RESULTS** | | |  |
| Study selection | 17 | Give numbers of studies screened, assessed for eligibility, and included in the review, with reasons for exclusions at each stage, ideally with a flow diagram. | 12-13, and Figure 1 |
| Study characteristics | 18 | For each study, present characteristics for which data were extracted (e.g., study size, PICOS, follow-up period) and provide the citations. | 13, and Table 1 |
| Risk of bias within studies | 19 | Present data on risk of bias of each study and, if available, any outcome level assessment (see item 12). | Supplementary Table 3 |
| Results of individual studies | 20 | For all outcomes considered (benefits or harms), present, for each study: (a) simple summary data for each intervention group (b) effect estimates and confidence intervals, ideally with a forest plot. | 14-15, Only topic a, Table 1 |
| Synthesis of results | 21 | Present results of each meta-analysis done, including confidence intervals and measures of consistency. | 16, Figures 2-4 |
| Risk of bias across studies | 22 | Present results of any assessment of risk of bias across studies (see Item 15). | 16-17, Figures 5-7 |
| Additional analysis | 23 | Give results of additional analyses, if done (e.g., sensitivity or subgroup analyses, meta-regression [see Item 16]). | 17, Figure 8 |
| **DISCUSSION** | | |  |
| Summary of evidence | 24 | Summarize the main findings including the strength of evidence for each main outcome; consider their relevance to key groups (e.g., healthcare providers, users, and policy makers). | 17-21; relevance by key groups is not reported |
| Limitations | 25 | Discuss limitations at study and outcome level (e.g., risk of bias), and at review-level (e.g., incomplete retrieval of identified research, reporting bias). | 21-22 |
| Conclusions | 26 | Provide a general interpretation of the results in the context of other evidence, and implications for future research. | 22-23 |
| **FUNDING** | | |  |
| Funding | 27 | Describe sources of funding for the systematic review and other support (e.g., supply of data); role of funders for the systematic review. | 2 |

**Supplementary Table 2.** Literature Search Strategy

| **Source** | **Search Phrase** | **Filters** |
| --- | --- | --- |
| PubMed | (((exercise[MeSH Terms] OR motor activity[MeSH Terms] OR exercise therapy[MeSH Terms] OR Circuit-Based Exercise[MeSH Terms]) AND (dementia OR alzheimer's disease OR vascular dementia OR Neurodegenerative Disease OR Neurocognitive disorder)) AND (cognition[MeSH Terms] OR activities of daily living[MeSH Terms] OR physical fitness[MeSH Terms] OR Physical Conditioning, Human[MeSH Terms] OR functional performance OR functional independence OR functional autonomy OR cognitive performance OR strength OR cardiorespiratory OR balance OR mobility OR flexibility OR attention OR executive function OR memory OR language OR perceptual-motor abilities)) NOT animals study | English  Humans  Clin Trials  Title/abstract |
| Web of Science | TS=((((“Exercise” OR “Exercises” OR “Physical Activity” OR “Activities, Physical” OR “Activity, Physical” OR “Physical Activities” OR “Exercise, Physical” OR “Exercises, Physical” OR “Physical Exercise” OR “Physical Exercises” OR “Acute Exercise” OR “Acute Exercises” OR “Exercise, Acute” OR “Exercises, Acute” OR “Exercise, Isometric” OR “Exercises, Isometric” OR “Isometric Exercises” OR “Isometric Exercise” OR “Exercise, Aerobic” OR “Aerobic Exercise” OR “Aerobic Exercises” OR “Exercises, Aerobic” OR “Exercise Training” OR “Exercise Trainings” OR “Training, Exercise” OR “Trainings, Exercise” OR “Motor Activity” OR “Activities, Motor” OR “Activity, Motor” OR “Motor Activities” OR “Exercise therapy” OR “Therapy, Exercise” OR “Exercise Therapies” OR “Therapies, Exercise” OR “Rehabilitation Exercise” OR “Exercise, Rehabilitation” OR “Exercises, Rehabilitation” OR “Rehabilitation Exercises” OR “Remedial Exercise” OR “Exercise, Remedial” OR “Exercises, Remedial” OR “Remedial Exercises” OR “Circuit-Based Exercise” OR “Circuit Based Exercise” OR “Circuit-Based Exercises” OR “Exercise, Circuit-Based” OR “Exercises, Circuit-Based” OR “Circuit Training” OR “Training, Circuit”) AND (“Dementia” OR “Dementias OR “Alzheimer disease” OR “Alzheimer's Disease” OR “Dementia, Senile” OR “Senile Dementia” OR “Alzheimer Syndrome” OR “Alzheimer Dementia” OR “Alzheimer Dementias” OR “Dementia, Alzheimer” OR “Dementias, Alzheimer” OR “Dementia vascular” OR “Dementias, Vascular” OR “Vascular Dementias” OR “Vascular Dementia” OR “Neurodegenerative Diseases” OR “Neurodegenerative Disease” OR Neurocognitive disorders” OR “Disorder, Neurocognitive” OR “Disorders, Neurocognitive” OR “Neurocognitive Disorder”)) AND (“Cognition” OR “Cognitions” OR “Cognitive Function” OR “Cognitive Functions” OR “Function, Cognitive” OR “Functions, Cognitive” OR “Activities of daily living” OR “ADL” OR “Activities, Daily Living” OR “Activity, Daily Living” OR “Daily Living Activities” OR “Daily Living Activity” OR “Living Activities, Daily” OR “Living Activity, Daily” OR “Limitation of Activity, Chronic” OR “Chronic Limitation of Activity” OR “Physical fitness” OR “Fitness, Physical” OR “Physical Conditioning, Human” OR “Conditioning, Human Physical” OR “Human Physical Conditioning” OR “Physical Training, Human” OR “Human Physical Training” OR “Training, Human Physical” OR “Functional performance” OR “functional independence” OR “functional autonomy” OR “cognitive performance” OR “strength” OR “cardiorespiratory” OR “balance” OR “mobility” OR “flexibility” OR “attention” OR “executive function” OR “memory” OR “language” OR “perceptual-motor abilities”)) NOT “animals study”) | English  Article  Not Review |
| SCOPUS | TITLE-ABS-KEY ((((Exercise OR “Physical Activity” OR “Physical Exercise” OR Acute OR Isometric OR Aerobic OR Training OR Motor OR Therapy OR Rehabilitation OR Remedial OR Circuit-Based Exercise OR Training) AND (Dementia OR Alzheimer OR Senile OR Vascular OR Neurodegenerative OR Neurocognitive)) AND (Cognition OR Cognitive OR “Activities of daily living” OR “ADL” OR Daily Living OR Fitness OR Physical Conditioning OR Physical Training OR “Functional performance” OR “functional independence” OR “functional autonomy” OR “cognitive performance” OR strength OR cardiorespiratory OR balance OR mobility OR flexibility OR attention OR “executive function” OR memory OR language OR “perceptual-motor abilities”)) AND NOT “animals study”) | English  Article  Human |
| SPORTSDISCUSS | (((“Exercise” OR “Exercises” OR “Physical Activity” OR “Activities, Physical” OR “Activity, Physical” OR “Physical Activities” OR “Exercise, Physical” OR “Exercises, Physical” OR “Physical Exercise” OR “Physical Exercises” OR “Acute Exercise” OR “Acute Exercises” OR “Exercise, Acute” OR “Exercises, Acute” OR “Exercise, Isometric” OR “Exercises, Isometric” OR “Isometric Exercises” OR “Isometric Exercise” OR “Exercise, Aerobic” OR “Aerobic Exercise” OR “Aerobic Exercises” OR “Exercises, Aerobic” OR “Exercise Training” OR “Exercise Trainings” OR “Training, Exercise” OR “Trainings, Exercise” OR “Motor Activity” OR “Activities, Motor” OR “Activity, Motor” OR “Motor Activities” OR “Exercise therapy” OR “Therapy, Exercise” OR “Exercise Therapies” OR “Therapies, Exercise” OR “Rehabilitation Exercise” OR “Exercise, Rehabilitation” OR “Exercises, Rehabilitation” OR “Rehabilitation Exercises” OR “Remedial Exercise” OR “Exercise, Remedial” OR “Exercises, Remedial” OR “Remedial Exercises” OR “Circuit-Based Exercise” OR “Circuit Based Exercise” OR “Circuit-Based Exercises” OR “Exercise, Circuit-Based” OR “Exercises, Circuit-Based” OR “Circuit Training” OR “Training, Circuit”) AND (“Dementia” OR “Dementias OR “Alzheimer disease” OR “Alzheimer's Disease” OR “Dementia, Senile” OR “Senile Dementia” OR “Alzheimer Syndrome” OR “Alzheimer Dementia” OR “Alzheimer Dementias” OR “Dementia, Alzheimer” OR “Dementias, Alzheimer” OR “Dementia vascular” OR “Dementias, Vascular” OR “Vascular Dementias” OR “Vascular Dementia” OR “Neurodegenerative Diseases” OR “Neurodegenerative Disease” OR Neurocognitive disorders” OR “Disorder, Neurocognitive” OR “Disorders, Neurocognitive” OR “Neurocognitive Disorder”)) AND (“Cognition” OR “Cognitions” OR “Cognitive Function” OR “Cognitive Functions” OR “Function, Cognitive” OR “Functions, Cognitive” OR “Activities of daily living” OR “ADL” OR “Activities, Daily Living” OR “Activity, Daily Living” OR “Daily Living Activities” OR “Daily Living Activity” OR “Living Activities, Daily” OR “Living Activity, Daily” OR “Limitation of Activity, Chronic” OR “Chronic Limitation of Activity” OR “Physical fitness” OR “Fitness, Physical” OR “Physical Conditioning, Human” OR “Conditioning, Human Physical” OR “Human Physical Conditioning” OR “Physical Training, Human” OR “Human Physical Training” OR “Training, Human Physical” OR “Functional performance” OR “functional independence” OR “functional autonomy” OR “cognitive performance” OR “strength” OR “cardiorespiratory” OR “balance” OR “mobility” OR “flexibility” OR “attention” OR “executive function” OR “memory” OR “language” OR “perceptual-motor abilities”)) NOT “animals study”) | English  Scientific Periodicals |

**Supplementary Table 3.** Methodological Quality of the Studies: TESTEX Scale Results

| **Study** | **1** | **2** | **3** | **4** | **5** | **6** | **7** | **8** | **9** | **10** | **11** | **12** | **Scores** |
| --- | --- | --- | --- | --- | --- | --- | --- | --- | --- | --- | --- | --- | --- |
| Barreto et al. (2017) | 1 | 1 | 1 | 1 | 0 | 3 | 1 | 2 | 1 | 0 | 0 | 1 | 12 |
| Burge et al. (2016) | 1 | 1 | 1 | 1 | 1 | 2 | 1 | 2 | 1 | 0 | 0 | 1 | 12 |
| Rolland et al. (2007) | 1 | 1 | 1 | 1 | 1 | 2 | 1 | 2 | 1 | 0 | 0 | 1 | 12 |
| Sampaio et al. (2016) | 1 | 0 | 0 | 1 | 0 | 1 | 1 | 2 | 1 | 0 | 1 | 1 | 9 |
| Steinberg et al. (2009) | 1 | 1 | 0 | 0 | 1 | 2 | 1 | 2 | 1 | 0 | 0 | 0 | 9 |
| Vreugdenhil et al. (2012) | 1 | 1 | 0 | 1 | 1 | 1 | 1 | 2 | 1 | 0 | 0 | 0 | 9 |

1) Eligibility of the participants; 2) Random distribution; 3) Allocation concealment of all participants at the time of randomization; 4) Comparison of groups at baseline; 5) Blinding of evaluators; 6) Measurement of at least one key outcome in 85% of subjects allocated; 7) Intention to treat analysis; 8) Comparison between groups; 9) Measures of accuracy and variability; 10) Activity monitored in control groups ; 11) Constant relative exercise intensity; 12) Exercise volume and energy expenditu
